# 4-(4-Nitro­phenoxy)biphen­yl

**DOI:** 10.1107/S1600536809007417

**Published:** 2009-03-06

**Authors:** Zareen Akhter, Toheed Akhter, Michael Bolte, M. Amin Baig, Humaira M. Siddiqi

**Affiliations:** a Department of Chemistry, Quaid-I-Azam University, Islamabad 45320, Pakistan; b Institut für Anorganische Chemie, J. W. Goethe-Universität Frankfurt, Max-von-Laue-Str. 7, 60438 Frankfurt/Main, Germany

## Abstract

The two phenyl rings of the biphenyl unit of the title compound, C_18_H_13_NO_3_, are almost coplanar [dihedral angle 6.70 (9)°]. The nitro­phenyl ring, on the other hand, is significantly twisted out of the plane of the these two rings, making dihedral angles of 68.83 (4)° with the middle ring and 62.86 (4)° with the end ring. The nitro group is twisted by 12.1 (2)° out of the plane of the phenyl ring to which it is attached.

## Related literature

The title compound is a precursor of amine which is a useful curing agent of ep­oxy resins. For the properties and applications of ep­oxy resins, see: Boey & Yap (2001 ▶); Bonnaud *et al.* (2004 ▶); de Moris *et al.* (2007 ▶); Van de Grampel *et al.* (2005 ▶); Agag & Takeichi (1999 ▶); Kagathera & Parsania (2001 ▶); Kagathera & Parsania (2001 ▶).
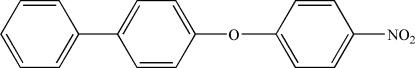


## Experimental

### 

#### Crystal data

C_18_H_13_NO_3_
*M* *_r_* = 291.29
Monoclinic, 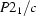

*a* = 9.6435 (7) Å
*b* = 5.8648 (3) Å
*c* = 24.6884 (18) Å
β = 95.704 (6)°
*V* = 1389.39 (16) Å^3^
*Z* = 4
Mo *K*α radiation
μ = 0.10 mm^−1^
*T* = 173 K
0.44 × 0.37 × 0.13 mm

#### Data collection

STOE IPDS II two-circle-diffractometer
Absorption correction: none
16020 measured reflections
2556 independent reflections
2187 reflections with *I* > 2σ(*I*)
*R*_int_ = 0.052

#### Refinement

*R*[*F* ^2^ > 2σ(*F* ^2^)] = 0.040
*wR*(*F* ^2^) = 0.118
*S* = 1.07
2556 reflections
200 parameters
H-atom parameters constrained
Δρ_max_ = 0.24 e Å^−3^
Δρ_min_ = −0.20 e Å^−3^

### 

Data collection: *X-AREA* (Stoe & Cie, 2001 ▶); cell refinement: *X-AREA*; data reduction: *X-AREA*; program(s) used to solve structure: *SHELXS97* (Sheldrick, 2008 ▶); program(s) used to refine structure: *SHELXL97* (Sheldrick, 2008 ▶); molecular graphics: *XP* in *SHELXTL-Plus* (Sheldrick, 2008 ▶); software used to prepare material for publication: *SHELXL97* and *PLATON* (Spek, 2009 ▶).

## Supplementary Material

Crystal structure: contains datablocks I, Za12. DOI: 10.1107/S1600536809007417/ww2140sup1.cif

Structure factors: contains datablocks I. DOI: 10.1107/S1600536809007417/ww2140Isup2.hkl

Additional supplementary materials:  crystallographic information; 3D view; checkCIF report

## Figures and Tables

**Fig. 1.**
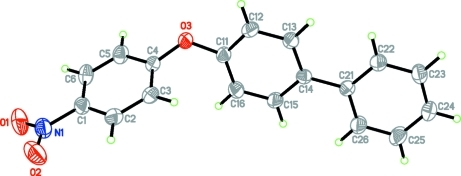
Perspective view of the title compound with the atom numbering scheme; displacement ellipsoids are at the 50% probability level.

**Fig. 2.**
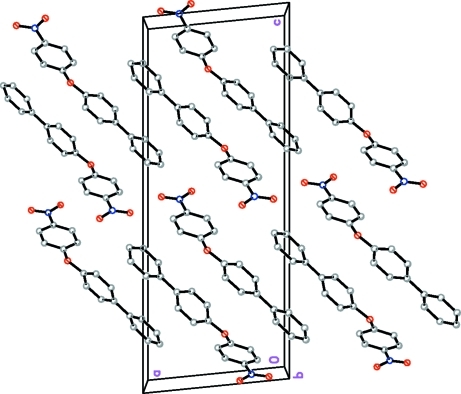
Packing of the title compound with view onto the ac plane, hydrogen atoms are omitted for clarity.

## supplementary crystallographic information

### Comment

Epoxy resins are a versatile group of crosslinked polymers that has excellent chemical resistance, good electrical insulating properties, good adhesion to glass and metal and can be easily fabricated (Boey & Yap, 2001). Variety of properties helps epoxy resins to meet performance requirements of some demanding applications (Bonnaud *et al.*, 2004). These include areas as diverse as construction, electronics, adhesives and coatings (de Moris *et al.*, 2007). The usefulness of epoxy resins is often limited due to their inherent brittleness arising from crosslinking structure (van de Grampel *et al.*, 2005). Development of approaches for toughening epoxy resins without sacrificing modulus and glass transition temperature (Tg) would lead to an increase in their applications (Kagathera & Parsania, 2001). One such approach is the curing of epoxy resins with different curing agents (Agag & Takeichi, 1999). The title compound is a precursor of amine which is a useful curing agent of epoxy resins.

The two phenyl rings of the biphenyl moiety of the title compound are almost coplanar [dihedral angle 6.70 (9)°]. The nitrophenyl ring, on the other hand, is significantly twisted out of the plane of the these two rings [68.83 (4)° and 62.86 (4)°]. The nitro group is twisted by 12.1 (2)° out of the plane of the phenyl ring to which it is attached.

### Experimental

A 500 ml two neck round bottom flask was equipped with condenser and thermometer and was charged with (0.059 moles) biphenyl-4-ol, (0.059 moles) anhydrous potassium carbonate and (0.059 moles) 4-chloronitrobenzene in 180 ml of DMF. Reaction mixture was heated for 24 h at 120°C. The reaction was carried out in the inert atmosphere of nitrogen. Progress of reaction was measured by TLC [1:1, ethyl acetae, n-hexane]. After completion, the reaction mixture was poured into 600 ml of water to give yellow precipitates. These precipitates were collected by filtration and washed with water several times. Recrystallization of the residue in n-hexane afforded the title compound (86%) (m.p 142–144°C)

### Refinement

H atoms were located in a difference map, but geometrically positioned and refined using a riding model with fixed individual displacement parameters [*U*_iso_(H) = 1.2 *U*_eq_(C)] and with C—H = 0.95Å.

### Crystal data

**Table:**

C_18_H_13_NO_3_	*F*(000) = 608
*M**_r_* = 291.29	*D*_x_ = 1.393 Mg m^−^^3^
Monoclinic, *P*2_1_/*c*	Mo *K*α radiation, λ = 0.71073 Å
Hall symbol: -P 2ybc	Cell parameters from 16639 reflections
*a* = 9.6435 (7) Å	θ = 2.2–25.8°
*b* = 5.8648 (3) Å	µ = 0.10 mm^−^^1^
*c* = 24.6884 (18) Å	*T* = 173 K
β = 95.704 (6)°	Plate, colourless
*V* = 1389.39 (16) Å^3^	0.44 × 0.37 × 0.13 mm
*Z* = 4	

### Data collection

**Table:**

STOE IPDS II two-circle-diffractometer	2187 reflections with *I* > 2σ(*I*)
Radiation source: fine-focus sealed tube	*R*_int_ = 0.052
graphite	θ_max_ = 25.5°, θ_min_ = 2.1°
ω scans	*h* = −11→11
16020 measured reflections	*k* = −7→6
2556 independent reflections	*l* = −29→29

### Refinement

**Table:**

Refinement on *F*^2^	Secondary atom site location: difference Fourier map
Least-squares matrix: full	Hydrogen site location: inferred from neighbouring sites
*R*[*F*^2^ > 2σ(*F*^2^)] = 0.040	H-atom parameters constrained
*wR*(*F*^2^) = 0.118	*w* = 1/[σ^2^(*F*_o_^2^) + (0.0735*P*)^2^ + 0.2032*P*] where *P* = (*F*_o_^2^ + 2*F*_c_^2^)/3
*S* = 1.07	(Δ/σ)_max_ < 0.001
2556 reflections	Δρ_max_ = 0.24 e Å^−^^3^
200 parameters	Δρ_min_ = −0.20 e Å^−^^3^
0 restraints	Extinction correction: *SHELXL*, Fc^*^=kFc[1+0.001xFc^2^λ^3^/sin(2θ)]^-1/4^
Primary atom site location: structure-invariant direct methods	Extinction coefficient: 0.028 (4)

### Special details

**Table:**

Geometry. All e.s.d.'s (except the e.s.d. in the dihedral angle between two l.s. planes) are estimated using the full covariance matrix. The cell e.s.d.'s are taken into account individually in the estimation of e.s.d.'s in distances, angles and torsion angles; correlations between e.s.d.'s in cell parameters are only used when they are defined by crystal symmetry. An approximate (isotropic) treatment of cell e.s.d.'s is used for estimating e.s.d.'s involving l.s. planes.
Refinement. Refinement of *F*^2^ against ALL reflections. The weighted *R*-factor *wR* and goodness of fit *S* are based on *F*^2^, conventional *R*-factors *R* are based on *F*, with *F* set to zero for negative *F*^2^. The threshold expression of *F*^2^ > σ(*F*^2^) is used only for calculating *R*-factors(gt) *etc*. and is not relevant to the choice of reflections for refinement. *R*-factors based on *F*^2^ are statistically about twice as large as those based on *F*, and *R*- factors based on ALL data will be even larger.

### Fractional atomic coordinates and isotropic or equivalent isotropic displacement parameters (Å^2^)

**Table:**

	*x*	*y*	*z*	*U*_iso_*/*U*_eq_	
N1	0.24460 (14)	1.3101 (2)	0.49386 (5)	0.0439 (3)	
O1	0.11760 (13)	1.3217 (2)	0.48231 (5)	0.0630 (4)	
O2	0.32703 (14)	1.4402 (2)	0.47506 (5)	0.0648 (4)	
O3	0.44070 (9)	0.60894 (15)	0.63327 (4)	0.0357 (3)	
C1	0.29854 (14)	1.1306 (2)	0.53120 (5)	0.0342 (3)	
C2	0.43662 (14)	1.1370 (2)	0.55251 (5)	0.0369 (3)	
H2	0.4960	1.2569	0.5431	0.044*	
C3	0.48706 (13)	0.9659 (2)	0.58781 (5)	0.0335 (3)	
H3	0.5816	0.9675	0.6030	0.040*	
C4	0.39846 (12)	0.7919 (2)	0.60091 (5)	0.0289 (3)	
C5	0.26016 (13)	0.7870 (3)	0.57875 (5)	0.0360 (3)	
H5	0.2008	0.6661	0.5876	0.043*	
C6	0.20939 (13)	0.9577 (3)	0.54400 (5)	0.0379 (3)	
H6	0.1148	0.9570	0.5290	0.045*	
C11	0.55429 (12)	0.6296 (2)	0.67283 (5)	0.0293 (3)	
C12	0.65196 (14)	0.4576 (2)	0.67578 (6)	0.0378 (3)	
H12	0.6466	0.3408	0.6490	0.045*	
C13	0.75837 (14)	0.4557 (2)	0.71807 (5)	0.0369 (3)	
H13	0.8249	0.3358	0.7200	0.044*	
C14	0.76992 (12)	0.6256 (2)	0.75787 (5)	0.0261 (3)	
C15	0.66981 (13)	0.7987 (2)	0.75286 (5)	0.0338 (3)	
H15	0.6755	0.9182	0.7790	0.041*	
C16	0.56253 (14)	0.8018 (2)	0.71109 (5)	0.0358 (3)	
H16	0.4955	0.9211	0.7088	0.043*	
C21	0.88361 (12)	0.6232 (2)	0.80367 (5)	0.0268 (3)	
C22	0.97567 (14)	0.4414 (2)	0.81201 (6)	0.0383 (3)	
H22	0.9662	0.3143	0.7881	0.046*	
C23	1.08153 (15)	0.4416 (3)	0.85470 (6)	0.0418 (4)	
H23	1.1432	0.3153	0.8594	0.050*	
C24	1.09758 (13)	0.6225 (2)	0.89004 (5)	0.0353 (3)	
H24	1.1693	0.6218	0.9194	0.042*	
C25	1.00812 (16)	0.8051 (3)	0.88227 (6)	0.0470 (4)	
H25	1.0183	0.9317	0.9063	0.056*	
C26	0.90334 (16)	0.8054 (3)	0.83963 (6)	0.0450 (4)	
H26	0.8432	0.9337	0.8348	0.054*	

### Atomic displacement parameters (Å^2^)

**Table:**

	*U*^11^	*U*^22^	*U*^33^	*U*^12^	*U*^13^	*U*^23^
N1	0.0575 (8)	0.0410 (7)	0.0321 (6)	0.0100 (6)	−0.0010 (5)	0.0015 (5)
O1	0.0585 (8)	0.0712 (9)	0.0559 (7)	0.0191 (6)	−0.0119 (6)	0.0174 (6)
O2	0.0780 (8)	0.0492 (7)	0.0662 (8)	−0.0006 (6)	0.0028 (6)	0.0237 (6)
O3	0.0332 (5)	0.0306 (5)	0.0403 (5)	−0.0062 (4)	−0.0109 (4)	0.0041 (4)
C1	0.0421 (7)	0.0337 (7)	0.0261 (6)	0.0059 (6)	0.0007 (5)	−0.0013 (5)
C2	0.0411 (7)	0.0324 (7)	0.0365 (7)	−0.0064 (6)	0.0002 (6)	−0.0005 (5)
C3	0.0287 (6)	0.0340 (7)	0.0365 (7)	−0.0041 (5)	−0.0030 (5)	−0.0016 (5)
C4	0.0298 (6)	0.0298 (7)	0.0263 (6)	0.0001 (5)	−0.0015 (5)	−0.0017 (5)
C5	0.0284 (6)	0.0446 (8)	0.0344 (6)	−0.0070 (5)	−0.0001 (5)	0.0049 (6)
C6	0.0288 (6)	0.0523 (9)	0.0316 (6)	0.0024 (6)	−0.0019 (5)	0.0036 (6)
C11	0.0264 (6)	0.0301 (7)	0.0304 (6)	−0.0044 (5)	−0.0017 (5)	0.0029 (5)
C12	0.0394 (7)	0.0325 (7)	0.0395 (7)	0.0034 (6)	−0.0067 (6)	−0.0114 (6)
C13	0.0349 (7)	0.0327 (7)	0.0410 (7)	0.0094 (5)	−0.0062 (6)	−0.0090 (6)
C14	0.0250 (6)	0.0256 (6)	0.0279 (6)	−0.0012 (5)	0.0038 (5)	−0.0004 (4)
C15	0.0343 (7)	0.0324 (7)	0.0338 (6)	0.0055 (5)	−0.0017 (5)	−0.0084 (5)
C16	0.0329 (7)	0.0340 (7)	0.0393 (7)	0.0092 (5)	−0.0020 (5)	−0.0032 (5)
C21	0.0247 (6)	0.0286 (6)	0.0272 (6)	−0.0013 (5)	0.0039 (5)	−0.0003 (5)
C22	0.0397 (7)	0.0352 (8)	0.0380 (7)	0.0075 (6)	−0.0052 (6)	−0.0087 (6)
C23	0.0387 (7)	0.0421 (8)	0.0425 (7)	0.0112 (6)	−0.0071 (6)	−0.0016 (6)
C24	0.0301 (6)	0.0463 (8)	0.0285 (6)	−0.0018 (6)	−0.0020 (5)	0.0019 (5)
C25	0.0462 (8)	0.0482 (9)	0.0438 (8)	0.0063 (7)	−0.0098 (6)	−0.0187 (7)
C26	0.0442 (8)	0.0402 (8)	0.0471 (8)	0.0129 (6)	−0.0122 (6)	−0.0163 (6)

### Geometric parameters (Å, °)

**Table:**

N1—O2	1.2260 (17)	C13—C14	1.3962 (17)
N1—O1	1.2315 (17)	C13—H13	0.9500
N1—C1	1.4608 (17)	C14—C15	1.3978 (17)
O3—C4	1.3751 (15)	C14—C21	1.4948 (17)
O3—C11	1.3984 (15)	C15—C16	1.3864 (18)
C1—C2	1.3828 (19)	C15—H15	0.9500
C1—C6	1.386 (2)	C16—H16	0.9500
C2—C3	1.3851 (19)	C21—C22	1.3895 (18)
C2—H2	0.9500	C21—C26	1.3902 (18)
C3—C4	1.3897 (18)	C22—C23	1.3924 (19)
C3—H3	0.9500	C22—H22	0.9500
C4—C5	1.3906 (17)	C23—C24	1.373 (2)
C5—C6	1.376 (2)	C23—H23	0.9500
C5—H5	0.9500	C24—C25	1.376 (2)
C6—H6	0.9500	C24—H24	0.9500
C11—C12	1.3769 (18)	C25—C26	1.385 (2)
C11—C16	1.3798 (18)	C25—H25	0.9500
C12—C13	1.3893 (18)	C26—H26	0.9500
C12—H12	0.9500		
			
O2—N1—O1	123.04 (13)	C12—C13—H13	119.2
O2—N1—C1	118.95 (13)	C14—C13—H13	119.2
O1—N1—C1	118.00 (13)	C13—C14—C15	116.74 (11)
C4—O3—C11	120.29 (9)	C13—C14—C21	121.79 (11)
C2—C1—C6	121.93 (12)	C15—C14—C21	121.47 (11)
C2—C1—N1	119.31 (13)	C16—C15—C14	122.23 (12)
C6—C1—N1	118.77 (12)	C16—C15—H15	118.9
C1—C2—C3	118.97 (12)	C14—C15—H15	118.9
C1—C2—H2	120.5	C11—C16—C15	119.17 (12)
C3—C2—H2	120.5	C11—C16—H16	120.4
C2—C3—C4	119.52 (12)	C15—C16—H16	120.4
C2—C3—H3	120.2	C22—C21—C26	116.75 (12)
C4—C3—H3	120.2	C22—C21—C14	121.95 (11)
O3—C4—C3	123.69 (11)	C26—C21—C14	121.29 (11)
O3—C4—C5	115.44 (11)	C21—C22—C23	121.41 (12)
C3—C4—C5	120.76 (12)	C21—C22—H22	119.3
C6—C5—C4	119.87 (12)	C23—C22—H22	119.3
C6—C5—H5	120.1	C24—C23—C22	120.63 (13)
C4—C5—H5	120.1	C24—C23—H23	119.7
C5—C6—C1	118.94 (12)	C22—C23—H23	119.7
C5—C6—H6	120.5	C23—C24—C25	118.90 (12)
C1—C6—H6	120.5	C23—C24—H24	120.5
C12—C11—C16	120.49 (12)	C25—C24—H24	120.5
C12—C11—O3	117.24 (11)	C24—C25—C26	120.44 (13)
C16—C11—O3	121.95 (11)	C24—C25—H25	119.8
C11—C12—C13	119.74 (12)	C26—C25—H25	119.8
C11—C12—H12	120.1	C25—C26—C21	121.86 (13)
C13—C12—H12	120.1	C25—C26—H26	119.1
C12—C13—C14	121.62 (12)	C21—C26—H26	119.1
			
O2—N1—C1—C2	12.16 (19)	C11—C12—C13—C14	−0.5 (2)
O1—N1—C1—C2	−168.59 (13)	C12—C13—C14—C15	−0.5 (2)
O2—N1—C1—C6	−167.52 (13)	C12—C13—C14—C21	179.53 (12)
O1—N1—C1—C6	11.73 (19)	C13—C14—C15—C16	0.9 (2)
C6—C1—C2—C3	−0.2 (2)	C21—C14—C15—C16	−179.06 (12)
N1—C1—C2—C3	−179.88 (11)	C12—C11—C16—C15	−0.5 (2)
C1—C2—C3—C4	0.17 (19)	O3—C11—C16—C15	172.89 (11)
C11—O3—C4—C3	27.36 (17)	C14—C15—C16—C11	−0.5 (2)
C11—O3—C4—C5	−156.29 (11)	C13—C14—C21—C22	−6.20 (19)
C2—C3—C4—O3	176.54 (11)	C15—C14—C21—C22	173.79 (12)
C2—C3—C4—C5	0.38 (19)	C13—C14—C21—C26	172.90 (13)
O3—C4—C5—C6	−177.36 (12)	C15—C14—C21—C26	−7.11 (19)
C3—C4—C5—C6	−0.9 (2)	C26—C21—C22—C23	0.6 (2)
C4—C5—C6—C1	0.8 (2)	C14—C21—C22—C23	179.73 (12)
C2—C1—C6—C5	−0.3 (2)	C21—C22—C23—C24	0.2 (2)
N1—C1—C6—C5	179.37 (12)	C22—C23—C24—C25	−0.6 (2)
C4—O3—C11—C12	−133.96 (13)	C23—C24—C25—C26	0.3 (2)
C4—O3—C11—C16	52.42 (16)	C24—C25—C26—C21	0.5 (3)
C16—C11—C12—C13	1.0 (2)	C22—C21—C26—C25	−1.0 (2)
O3—C11—C12—C13	−172.73 (12)	C14—C21—C26—C25	179.89 (13)
